# Arthroscopic Anatomical Repair of Anterior Talofibular Ligament for Chronic Lateral Instability of the Ankle: Medium‐ and Long‐Term Functional Follow‐Up

**DOI:** 10.1111/os.12651

**Published:** 2020-03-02

**Authors:** Shi‐ming Feng, Qing‐qing Sun, Ai‐guo Wang, Bu‐qing Chang, Jian Cheng

**Affiliations:** ^1^ Hand and Foot Microsurgery Department, Xuzhou Central Hospital Xuzhou China; ^2^ Xuzhou Clinical College of Xuzhou Medical University Xuzhou China

**Keywords:** Anterior talofibular ligament, Anatomical repair, Chronic lateral ankle instability, Minimally invasive surgery

## Abstract

**Objective:**

To evaluate the functional outcomes of arthroscopic anatomical repair of anterior talofibular ligament (ATFL) in the treatment of chronic lateral ankle instability (CLAI) during medium‐ and long‐term follow‐up.

**Methods:**

From September 2014 to August 2017, the data of 37 patients (23 males, 14 females; 12 left ankles, 25 right ankles) aged between 21 and 56 years, with an average age of 32.17 ± 6.35 years, presenting with CLAI, was retrospectively analyzed. Among them, 32 injuries were caused by sprain and five injuries were caused by car accidents. The course of the disease lasted for 12 to 60 months, with an average of 26.07 ± 13.29 months. All patients had intact skin around the ankle and no skin lesions. All patients underwent arthroscopic anatomical repair of ATFL, with the fixation of one to two anchors. Pre‐ and post‐operative visual analogue scales (VAS), the American Orthopaedic Foot and Ankle Society Ankle‐Hindfoot Score (AOFAS), and the Karlsson Ankle Functional Score (KAFS) were compared to evaluate the curative effect of the operation.

**Results:**

The operation was successful in all 37 cases. The operation time ranged from 40 to 75 min, with an average of 51.25 ± 11.49 min. After surgery, all incisions healed in stage I and there were no complications such as nerve, blood vessel and tendon injury, implant rejection, or suture rejection. Hospital stays of postoperative patients were 3 to 5 days, with an average of 3.77 ± 1.36 days. All patients were followed for 24 to 45 months, averaging 33.16 ± 10.58 months. For three patients with CLAI combined with mild limitation of subjective ankle movement, joint activity was normal after rehabilitation function exercise and proprioceptive function training for 2 months. At the final follow‐up, ankle pain had disappeared completely. The ankle varus stress test and ankle anterior drawer test were both negative. Range of joint motion was good. There was no lateral instability of the ankle and all patients returned to normal gait. The mean VAS score decreased to 1.12 ± 0.13, the AOFAS score increased to 92.53 ± 4.87, and the KAFS score increased to 93.36 ± 6.15. All the follow‐up indexes were significantly different from those before surgery.

**Conclusion:**

Arthroscopic anatomical repair of ATFL for CLAI is precise, with less surgical trauma and reliable medium‐ and long‐term effect.

## Introduction

The lateral ligament of the ankle is a key ligament structure to maintain stability of the ankle, and consists of the anterior talofibular ligament (ATFL), calcaneofibular ligament (CFL), and posterior talofibular ligament (PTFL). Due to the anatomical and motor characteristics of the ankle, approximately 85% of the ankle sports injuries are varus injuries, which is damage to the lateral ligament structure, and of which 62% are combined with ATFL injury^1^, ^2^. Most of the patients with an ankle varus injury can obtain satisfactory results from conservative treatment for 3 to 6 months^3^, ^4^. However, 10% to 12% of patients still report lateral ankle pain, repeated ankle sprains, leg giving way when walking, and fear of walking at night for more than 6 months following injury, and this can develop into chronic lateral ankle instability (CLAI)^5^. CLAI is divided into symptomatic lateral ankle instability and mechanical lateral ankle instability, based on to the integrity of the lateral ankle ligament. If the structure of the symptomatic lateral ankle ligament is complete, satisfactory therapeutic effect can be obtained by proprioceptive exercise^6^. Mechanical lateral ankle instability is the true instability of the ankle due to rupture of the lateral ligament of the ankle, and the stability of the lateral ankle should be restored by operation^7^.

The current surgical treatment of CLAI is mainly divided into anatomical repair of the ligament and strengthening of the lateral ligament. In open surgery, the Broström procedure with direct ATFL repair and Broström‐Gould procedure combined with extensor retinaculum suture to strengthen the lateral ligament of the ankle are considered the gold standard procedures for the treatment of CLAI^8^, ^9^. The above surgical procedures are performed for treatment of CLAI in traditional open surgery. The traditional open surgery has been widely used for decades, for it has been proved to be an effective strategy with good surgical outcomes. However, the procedure requires a curved incision starting approximately 5 cm on the lateral ankle, and includes the drawbacks of a long surgical incision and large surgical trauma. With heightened requirements for postoperative aesthetic appearance and functional recovery, minimally invasive treatment of ankle sports injury is increasingly sought by both doctors and patients. With the development of arthroscopic technology in recent years, arthroscopic repair of the lateral ankle ligament has become one of the hotspots in foot and ankle surgery and sports medicine^10^. Through systematic analysis, Guelfi *et al*. compared the results of open surgery and arthroscopic surgery^11^. In the comparison of 505 cases involved in 13 open studies and 216 cases involved in six arthroscopic studies, it is found that both open surgery and arthroscopic surgery can obtain excellent efficacy for the treatment of CLAI. Although the incidence of postoperative complications after arthroscopic surgery is higher, the postoperative satisfaction of patients is significantly higher than that of traditional open surgery^11^. By comparing the data of 37 patients undergoing open Broström repair surgery and 23 patients undergoing arthroscopic Broström repair surgery, Li *et al*. found that there was no significant statistical difference in AOFAS, KAFS, and Tegner score between the two groups after a 2‐year follow‐up^12^. It is believed that arthroscopic surgery can achieve the same effect as open surgery. However, as a minimally invasive technology, arthroscopic surgery has less trauma and faster postoperative recovery. Batista *et al*. treated 22 patients with CLAI through the “All inside” lateral ligament repair procedure by anterior medial and anterolateral portals^13^. After 17–31 months of follow‐up, the AOFAS score increased from 63 points to 90 points and no CLAI recurrence was found after surgery. Batista *et al*. believed that the “All inside” lateral ligament repair procedure can obtain lower complications and lower local morbidity than the traditional open surgery, and should be the first‐stage procedure for surgical treatment of CLAI^13^. Through the above literature analysis, it can be seen that although there is no consensus on whether arthroscopic surgery or open surgery is more effective for CLAI, more and more scholars recommend the use of arthroscopic anatomical repair for CLAI. Under the condition that the same function and result can be obtained with open surgery, arthroscopic surgery is less invasive and more satisfying. However, the current literature reports mainly focus on the short‐term effect of objective direct repair to ATFL for CLAI^14^, ^15^, ^16^. There are seldom literature reports on the long‐term functional outcome.

The purpose of this present retrospective study was as follows. First, we aimed to investigate the therapeutic effect of arthroscopic anatomical repair of ATFL in the treatment of CLAI. Second, we evaluated whether the arthroscopic anatomical repair of ATFL for CLAI has a good medium‐ and long‐term surgical outcome through a follow‐up for at least 2 years. Third, we analyzed the complications, such as infection, nerve and tendon injury, and rejection. And we used the American Orthopaedic Foot and Ankle Society Ankle‐Hindfoot Score (AOFAS), the Karlsson Ankle Functional Score (KAFS), and other scoring criteria in an effort to provide a reference basis for the clinical application of this type of operation.

## Materials and Methods

This study was approved by the Hospital Ethics Committee and all selected patients provided signed informed consent.

### *Inclusion and Exclusion Criteria*

Inclusion criteria: (i) patients with chronic lateral ankle instability diagnosed by physical examination and imaging from September 2014 to August 2017; (ii) patients with regular conservative treatment for at least 6 months with no relief of ankle symptoms; (iii) patients who received arthroscopic anatomical repair with suitable residual tension and length for ATFL; and (iv) patients with avulsion injury of ATFL fibula side confirmed under the arthroscopy.

Exclusion criteria: (i) patients with symptomatic lateral instability of the ankle or instability of other joints (subtalar joint); (ii) patients with complicated diseases such as foot and ankle deformities, abnormal line of force, fracture, joint stiffness, or other ligament injuries; (iii) patients with complicated central and peripheral neuromuscular atrophy or ligament relaxation; (iv) patients who underwent ATFL reconstruction or had higher requirements for exercise^10^; (v) patients with abnormal bone structure around the insertion of the distal fibular ATFL ligament that could not be implanted with anchors; (vi) patients with osteoarthritis of the ankle or osteochondral injury requiring osteochondral transplantation; (vii) patients who had received follow‐up within less than 12 months or those with complicated, serious underlying diseases and could not tolerate the operation; (viii) patients who had complicated rupture of the calcaneofibular ligament or posterior talofibular ligament; and (ix) patients who had an avulsion fracture of lateral malleolus and a diameter of fracture block greater than 5 mm.

### *Participants*

We retrospectively analyzed data from 37 patients with CLAI (23 males, 14 females; 12 left ankles, 25 right ankles), aged between 21 to 56 with an average age of 32.17 ± 6.35 years. The Body Mass Index (BMI) was 22.69 ± 5.13 kg/m^2^ (range, 18.61 to 27.35 kg/m^2^). Thirty‐two of these injuries were caused by sprain and five injuries were caused by car accidents. The course of the disease lasted for 12 to 60 months, with an average of 26.07 ± 13.29 months. All patients had intact skin around the ankle and no skin lesions. The preoperative visual analogue scale (VAS), the American Orthopaedic Foot and Ankle Society (AOFAS) Ankle‐Hindfoot Score^17^, and Karlsson Ankle Functional Score (KAFS)^18^ were 4.79 ± 1.85, 73.16 ± 11.23 and 75.02 ± 9.37, respectively.

### *Surgical Technique*

#### *Anesthesia and Position*

In this group, nerve blocks of the lower extremity were used for operative anesthesia in 21 cases, intraspinal block anesthesia in 11 cases, and general intravenous anesthesia in five cases. The patient was placed in a supine position. A 7 cm‐high cushion was used on the hip of the affected side so that the affected foot was in the neutral position of the ankle under general anesthesia. The affected foot was placed on the distal edge of the operating table. An airbag tourniquet was placed mid‐thigh. After using tourniquet, the balloon pressure was set to 60 kPa.

#### *Approach, Exposure, and Arthroscopy Debridement*

**Figure 1:**
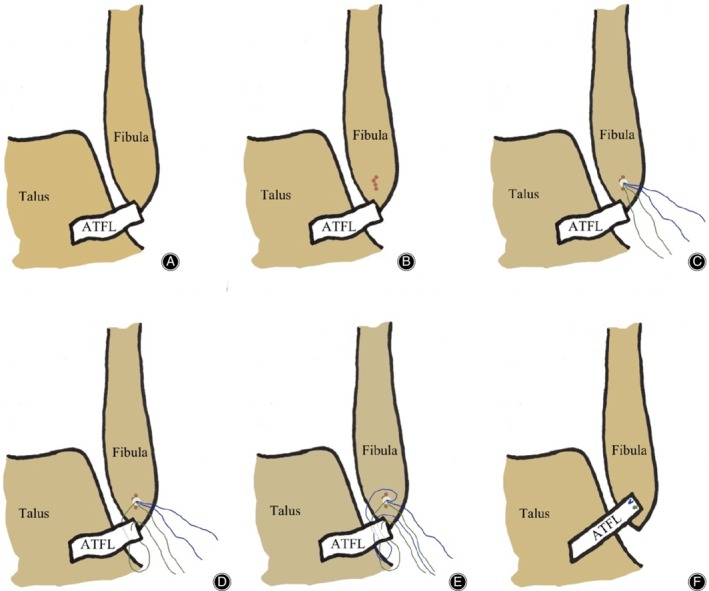
Surgical diagrams of arthroscopic anatomical repair of anterior talofibular ligament (ATFL). (A) The intact ATFL was avulsed from the fibula. (B) The bone of the fibula footprint was freshened using a Pituitary Rongeur or 1.0 mm Kirschner wire drill. (C) A double wire anchor with a diameter of 3.5 mm was inserted in the middle area of the fibula footprint. (D) The suture method of the first anchor sutural wire. (E) The suture method of the second anchor sutural wire. (F) The ATFL was sutured.

Twenty milliliters of saline was injected at the horizontal level of the ankle and 0.5 cm medial to the anterior tibial tendon to fill the articular cavity and dilate the articular capsule. A no. 11 scalpel blade was used to establish a medial approach of 0.5 cm anterior to the ankle at the same point. A mosquito hemostat was used to bluntly separate the entrance to the ankle cavity. The joint lens was inserted to explore the structure of the ankle cavity and anterior and lateral tissue of the ankle. Hyperplastic synovial tissue of the ankle, injured articular cartilage, and relaxed ATFL after avulsion was observed in most patients. A 0.5 cm incision was made at the level of the third peroneal muscle and lateral superficial peroneal nerve under the light source of the arthroscopic lens to establish an anterolateral approach to the ankle. A 4.5 or 3.5 mm shaver was inserted into the incision to clean the hyperplastic synovium, corpus liberum, and cartilage fragments in the ankle cavity. The condition of exfoliation and injury of talus cartilage was explored. Grinding was carried out according to each situation, and talus microfracture was performed. Should be thoroughly removed if the patients were combined with anterior ankle soft tissue impact and the condition was caused by the Bassett's ligament. Then, the arthroscope was introduced in to the ankle through the anterolateral portal. The stress test and the anterior drawer test of the ankle were performed under arthroscopy to reconfirm the lateral instability of the ankle. The anterolateral approach was made in parallell at 1 cm distal and 1 cm anterior to the tip of the lateral malleolus. A shaver was inserted to clean up the synovial and inflammatory tissue around the lateral malleolus. The condition of ATFL was explored. ATFL in all patients were avulsed from the peroneal side (Fig. 1A).

Flaccid and tension‐free ATFL and partial retraction of the fibula avulsion end were observed in the arthroscopic exploration. Two cases of small fractures with avulsion of the fibula were thoroughly cleared. During the operation, the residual proximal ATFL was clamped with a suture clamp. When it could be retracted to the footprint region of the distal fibula and the ligament was reliable and powerful, ATFL could be anatomically repaired. Otherwise, the Broström‐Gould procedure or ligament reconstruction was performed (not included in this study). The anterior fibula approach could be established according to the need of the operation for the safety of the anterolateral fibula.

#### *Anchor Insertion*

Under the arthroscopy, the bone of the fibula footprint was freshened using a Pituitary Rongeur or 1.0 mm Kirschner wire drill (Fig. 1B). A double‐wire anchor with a diameter of 3.5 mm was inserted in the middle area of the fibula footprint (Fig. 1C). The direction of the anchor was at an angle of 30°–45° to the *y*‐axis of the fibula. After clamping the proximal end of the ATFL, a certain tension of the ATFL should be maintained to explore the midpoint of the proximal region with relatively good structural quality of the ATFL as the needle insertion point.

#### *Repair the Ligament*

**Figure 2:**
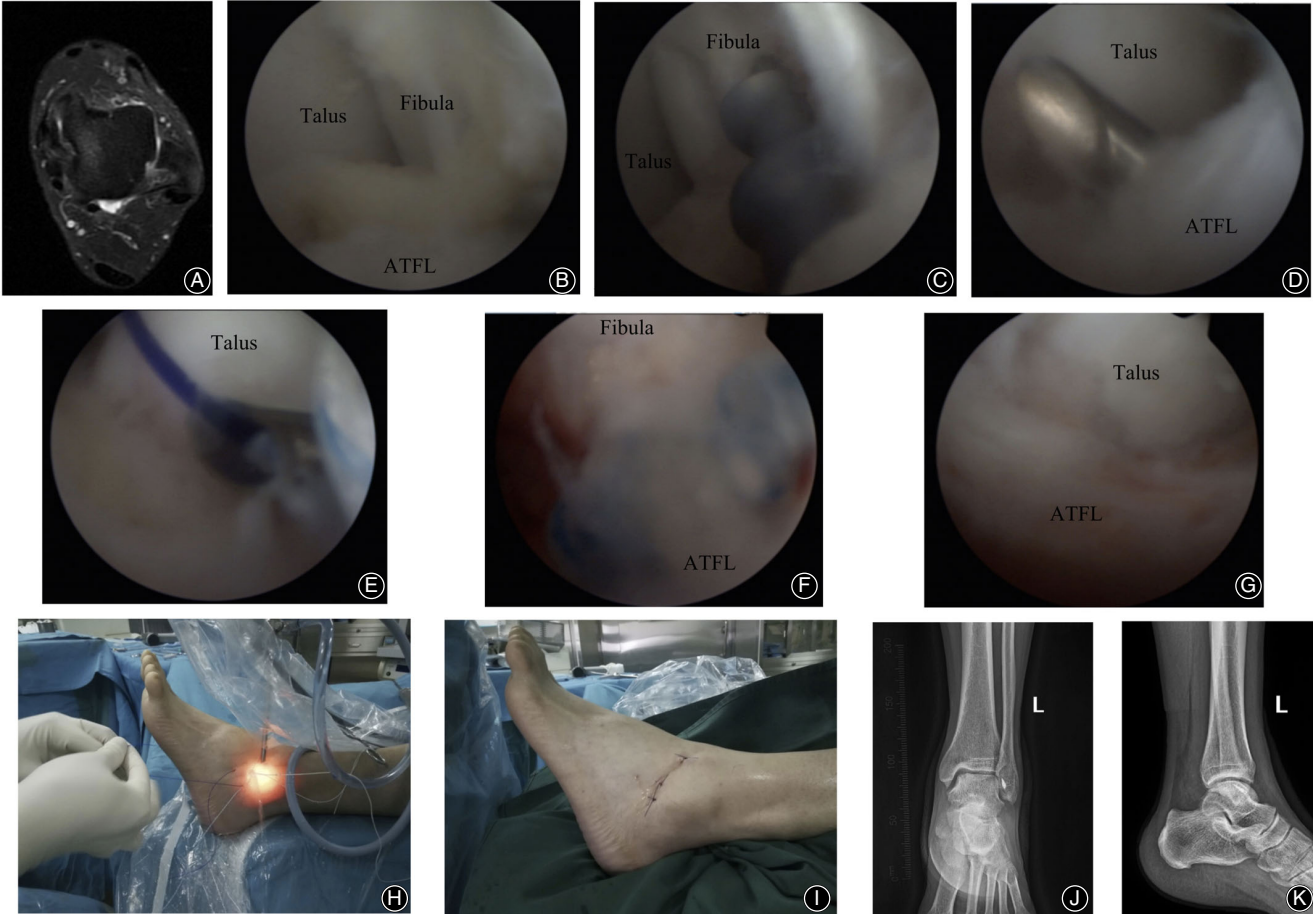
A 32‐year‐old male with recurrent sprain of the left ankle with unstable walking for 18 months. The symptoms were not relieved after 12 months of regular conservative treatment. Arthroscopic anatomical repair of ATFL was performed. (A) Preoperative MRI plain scan of the left ankle suggested that continuity of ATFL was interrupted. (B) Arthroscopic exploration showed horizontal avulsion of ATFL from the fibular stop point and relaxation of ATFL without tension. (C) The footprint region of the fibula was observed under arthroscopy. After freshening, a double wire anchor with a diameter of 3.5 mm was inserted. (D) The ATFL was sutured with a suture hook under arthroscopy. (E) Under arthroscopy, the anchor sutural line was guided through the ATFL after the PDS sutural line was used to pass through the suture hook. (F) Fibular side of the ATFL was sutured with a knot pusher. (G) The ATFL returned to normal tension and strength after suture under arthroscopy. (H) During the operation, the PDS sutural line was used to guide the anchor wire through the external phase. (I) The surgical approach after suture. (J, K) Anterior–posterior and lateral X‐ray films of the ankle after surgery.

**Figure 3:**
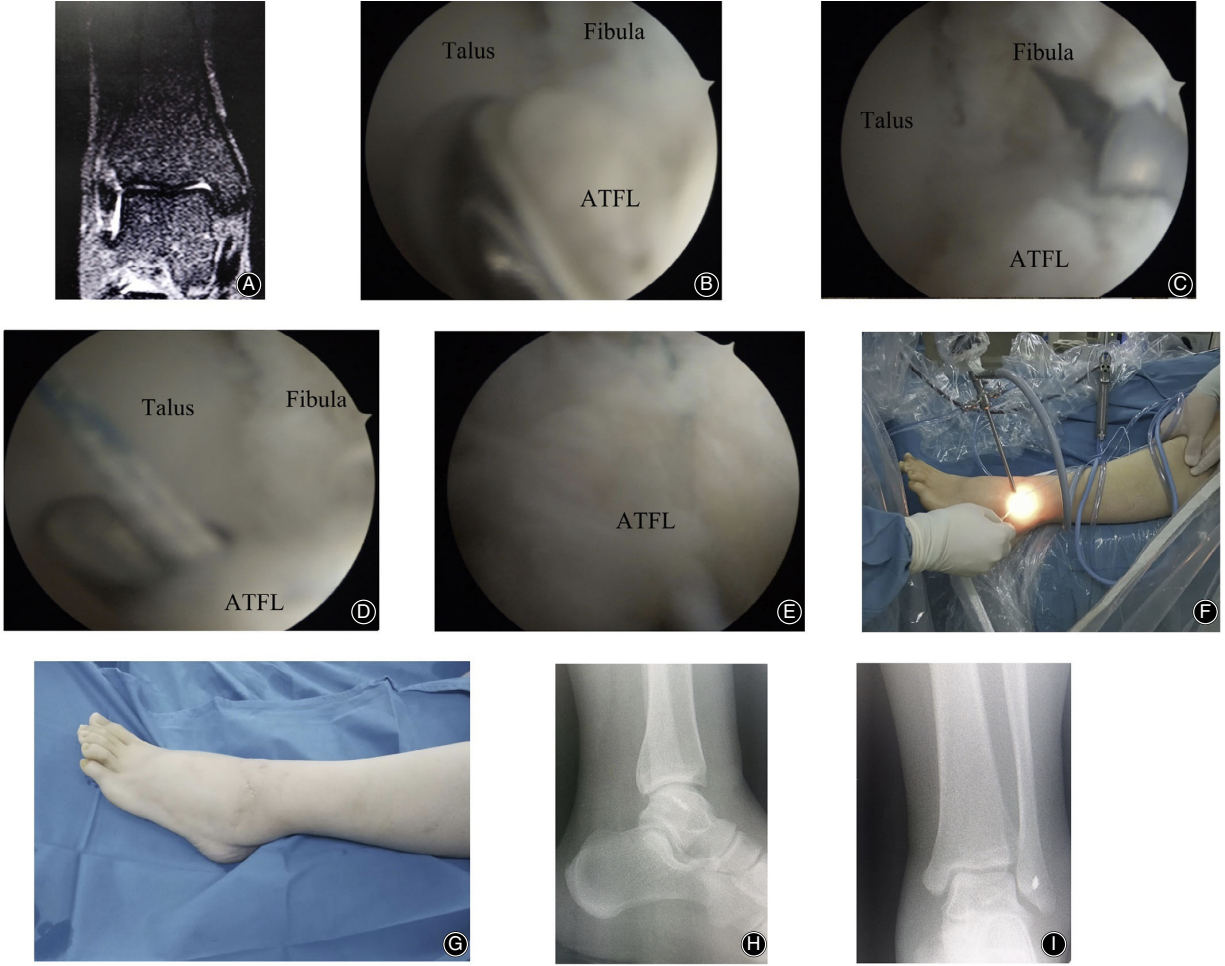
A 28‐year‐old female. The symptoms of CLAI were still presented after 8 months of conservative treatment. (A) Preoperative coronal MRI scan of the left ankle suggested the interruption of ATFL. (B) Flaccid and tension‐free ATFL that avulsed from the fibula point was observed under the arthroscopy. (C) A double wire anchor with a diameter of 3.5 mm was inserted into the fibular footprint region. (D) The ATFL was sutured with a suture hook under arthroscopy. (E) The ATFL was sutured and it returned to normal tension under arthroscopy. (F) External image of the arthroscopic procedure during the operation. (G) The portals of the surgery after the operation. (H) Lateral X‐ray of the ankle after surgery. (I) Anterior–posterior X‐ray of the ankle after surgery.

**Figure 4:**
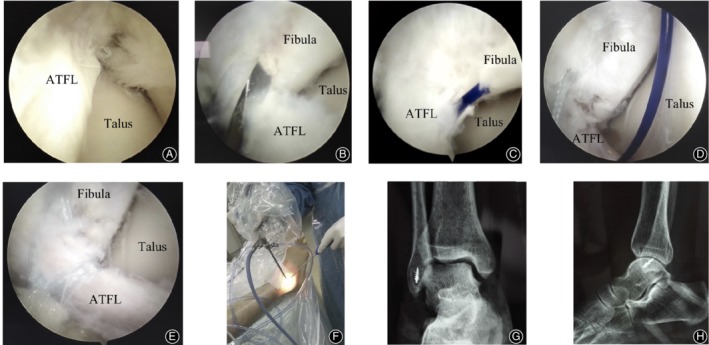
A 42‐year‐old male with the CLAI for 24 months. (A) Intra‐operative arthroscopic exploration showed the ATFL avulsion from the fibular. (B) The footprint region of the fibula was refreshed by using a 1.0 mm Kirschner wire drill. (C) Suture the first anchor sutural wire by using the PDS as a guider. (D) Suture of the second sutural wire guided by PDS. (E) The sutured ATFL under arthroscopy. (F) External image of the arthroscopic procedure during the operation. (G) Anterior–posterior X‐ray of the ankle after surgery. (H) Lateral X‐ray of the ankle after surgery.

The suture hook that had been pierced with the PDS sutural line was inserted. The PDS sutural line was led to the outside of the skin. A strand of the anchor sutural wire was introduced into the ligament and penetrated out of the skin through the PDS sutural line. The suture hook, which had been pierced with the PDS sutural line, was inserted at the vertical point of this position, which led to the same strand of anchor sutural line (Fig. 1D). The above suture method could be repeated once to strengthen the suture fixation. The threading of another suture wire on the anchor could be carried out in the same way (Fig. 1E). Finally, after confirming the strength of the suture under a microscope, the valgus ankle was in dorsiflexion. The knot pusher was used to suture one of the sutural lines and confirm the suture strength. The other sutural line was sutured (Fig. 1F). Under the microscope, the integrity and tension of the AFTL were again explored. The anterior drawer test and ankle varus stress test were performed again. After confirming the ligament suture effect, the arthroscopy was withdrawn and the approach incision was closed (typical cases are shown in Figs. 2, 3, 4).

### *Postoperative Management*

The ankle was fixed with a short leg brace in the position of slight dorsal extension and valgus. Antibiotics were routinely used within 24 h after surgery. On the second day after surgery, patients were advised to do early weight‐free functional and isometric exercises of their lower limb muscles. Removal of stitches was performed 2 weeks after operation. For patients with talus cartilage injury in microfracture, the brace remained fixed for 6 weeks after surgery; all other patients were fixed for 4 weeks. After 4 (or 6) weeks, ankle‐walking boots were used. Postoperative improvement in muscle strength around the ankle was assisted by the Rehabilitation Department. After 8 weeks, ankle range of motion, varus activity, and metatarsal flexion activity had increased. Twelve weeks after surgery, the patients were advised to gradually resume physical activity.

### *Postoperative Follow‐up and Observation Indexes*

Wound healing, ankle stability, and ankle function were evaluated using VAS, AOFAS, and KAFS scores. The last follow‐up time was when the patient was satisfied or unwilling to continue in the follow‐up.

### *Outcome Evaluation*

In order to better record the ankle function of patients, before scoring, all patients received the guidance of a professional physician so that they could better use the rating scale to reflect their real ankle function.

#### *Visual Analogue Scale (VAS)*

VAS scoring system is one of the most widely used rating scales to evaluate pain in clinic. The scores range from 0 to 10 points, with 0 points indicating no pain and 10 points indicating the most pain. The score results can be divided into the following three degrees: 0–3 points is considered mild pain that does not affect sleep; 4–6 points is considered moderate pain that affects sleep, but still allows sleep; 7–10 points is considered intense pain where the person is unable to sleep.

#### *American Orthopaedic Foot & Ankle Society Scale (AOFAS)*

AOFAS is the most commonly used scoring system for ankle function. The total score of the scoring system is 100 points, including three parts: pain (40 points), function (50 points), force line (10 points). Among them, the functional score includes the following seven parts: limited mobility, maximum walking distance, whether the road surface affects walking, gait, sagittal mobility, hind‐foot mobility, ankle and hind‐foot stability. The higher the score, the better the ankle function.

#### *Karlsson Ankle Function Score (KAFS)*

Karlsson ankle function scoring system is mainly used to evaluate the functional recovery after ankle surgery, which consists of eight parts: pain, swelling, subjective instability, rigidity, climbing the stairs, running, work and life, and the use of external aids. The total score of the scoring system is 100 points. The higher the score, the better the ankle function.

#### *Complications*

All complications were recorded, including infection, nerve injury, blood vessel injury, tendon injury, implant rejection or future rejection, ankle pain, ankle mobility, lateral ankle stability, and joint instability recurrence. The evaluation of surgical complications is of great significance to the feasibility and safety of the operation. Three experienced foot and ankle surgeons who are totally unaware of the operation and procedures evaluate the complications of the operation. If there are three different opinions, they must discuss them to reach the final conclusion.

### *Statistical Analysis*

SPSS (version 19.0, Chicago, IL, USA) statistical software was used to analyze the data. The quantitative data were expressed as mean ± standard deviation. Pre‐operative and postoperative group comparison of classified variables by *t*‐test or nonparametric test, and the correlation analysis of continuous variables by univariate analysis. The Spearman test was used to evaluate associations among functional outcomes of age, BMI, and disease duration. The α value was set as 0.05 due to the univariate comparisons before and after surgery. A *P* value < 0.05 was considered statistically significant.

## Results

### *Follow‐up*

All the patients were followed up in the attending physician's clinic department from the time they were discharged. Routine reexamination after surgery was performed in all patients, and the general circumstances and functional scores were recorded at the follow‐up time. The follow‐up time point and the information were collected at 6 months, 12 months, 24 months, 36 months, and 48 months after surgery. However, the final exact follow‐up time of all patients may not follow the above time, due to personal factors relating to patients. All patients were followed for 24 to 45 months, with an average of 33.16 ± 10.58 months.

### *General Results*

The operation was successful in all 37 cases. The operation time ranged from 40 to 75 min, with an average of 51.25 ± 11.49 min. All patients underwent ankle synovial tissue cleaning. During the operation, 19 patients underwent talus microfracture. Six patients had complicated Bassett's ligament anterior ankle impingement syndrome and the Bassett's ligament was removed. Resection of old small fractures of distal fibula was performed in two patients. The average postoperative hospital stay was 3.77 ± 1.36 days (interquartile range 3–5 days).

### *Clinical Improvement*

For three patients with CLAI combined with mild limitation of subjective ankle movement, joint activity was normal after rehabilitation function exercise and proprioceptive function training for 2 months. Ankle pain had disappeared completely. Ankle varus stress tests were negative. Ankle anterior drawer tests were negative. The range of motion of the patients was great, and all patients returned to a normal gait.

### *Implant Evaluation*

At the last follow‐up, there was no implant rejection or suture rejection. All anchors were fixed firmly without obvious signs of failure or evidence of detachment.

### *Functional Evaluation*

The VAS score was decreased to 1.12 ± 0.13 (*t* = 12.037, *P* = 0.00). The AOFAS score was increased to 92.53 ± 4.87 (*t* = 9.626, *P* = 0.00). The KAFS score was increased to 93.36 ± 6.15 (*t* = 9.953, *P* = 0.00). All the follow‐up indexes were significantly different from those before operation (Table 1). Subgroup analysis of all patients with CLAI revealed that the male patients resulted in similar functional outcomes to the female patients at the last follow‐up (VAS: 1.11 ± 0.17 *vs* 1.14 ± 0.20, *t* = 0.468, *P* = 0.644; AOFAS: 92.72 ± 5.01 *vs* 92.16 ± 4.23, *t* = −0.364, *P =* 0.719; KAFS: 93.65 ± 5.37 *vs* 93.08 ± 5.90, *t* = 0.295, *P* = 0.771). For patients with BMI lower 25.0 kg/m^2^ (*n* = 25), AOFAS (93.24 ± 3.37 *vs* 91.02 ± 2.74, *t* = 2.136, *P* = 0.042) and KAFS (94.35 ± 5.02 *vs* 91.11 ± 4.14, *t* = 2.076, *P* = 0.048) functional scores were better than that with BMI over 25.0 kg/m^2^ (*n* = 12). A negative correlation (nonlinear relationship) was found between AOFAS and BMI (Spearman correlation coefficient, −0.032; *P* = 0.546) and KAFS and BMI (Spearman correlation coefficient, −0.020; *P* = 0.759). Thus, a higher degree of AOFAS and KAFS will result if the BMI is lower.

**Table 1:** Functional evaluation (mean ± SD)

Evaluation Tools	Pre‐operation	Post‐operation	*t* value	*P* value
VAS	4.79 ± 1.85	1.12 ± 0.13	12.037	0.00
AOFAS	73.16 ± 11.23	92.53 ± 4.87	9.626	0.00
KAFS	75.02 ± 9.37	93.36 ± 6.15	9.953	0.00

AOFAS, American orthopedic foot and ankle society; KAFS, Karlsson ankle functional score; VAS, visual analogue scale. A value *P* < 0.05 was set as statistically significant.

### *Complications*

All incisions healed in stage I after surgery, and there were no complications such as wound infection, nerve injury, vascular injury, and tendon injury. At the final follow‐up, patients had no mechanical or symptomatic instability of the ankle. Good clinical outcomes in all the patients were observed without instance of ankle pain, joint stiffness, and arthritis until final follow‐up.

## Discussion

### *Surgical Management of CLAI*

Most cases of symptomatic lateral ankle instability can be treated with conservative treatment and rehabilitation exercise. However, surgical treatment is needed for mechanical lateral ankle instability. Because the residual ATFL ligament in CLAI is still of good quality, and the position of the ligament is superficial, the Broström procedure and modified Broström procedure for suturing extensor retinaculum have become the standard procedures for treatment of CLAI since Broström reported successful treatment of CLAI in 1996 by the direct repair of ATFL^19^, ^20^. Although the previous reports^21^, ^22^ indicated that use of the Broström procedure of direct repair of ATFL for CLAI has achieved satisfactory clinical results, there was still a 6% to 25% complication rate including ankle pain, ankle swelling, and recurrence of lateral ankle instability^23^. With the development of orthopaedic endoscopy, some clinicians adopted arthroscopy‐assisted ATFL repair, first using arthroscopy to deal with ankle and surrounding lesions, and then performing open suture repair for ATFL^24^. Liszka *et al*. found that most patient cases presenting with CLAI were complicated with medial talus cartilage injury, thus the traditional Broström procedure could not detect and deal with the above‐mentioned lesions^25^, ^26^. Arthroscopic exploration and debridement should be carried out before the incision of Broström procedure to repair ATFL^25^. In the current study, synovial tissue hyperplasia was found in all patients. Nineteen of 37 patients had talus cartilage injury and six patients had anterior soft tissue impingement syndrome of the ankle. Therefore, arthroscopy is beneficial to the comprehensive evaluation and treatment of ankle lesions. Song *et al*.^27^ compared arthroscopic ATFL repair with open repair of ATFL in 207 patients with CLAI and obtained the same ligament fixation strength and clinical effect. Arthroscopic repair of ATFL can comprehensively evaluate the ligament condition of ATFL, deal with the complicated lesions, and realize satisfactory anatomical repair^28^. Based on the clinical evaluation of this group, all 37 patients included in the study achieved satisfactory clinical results. The mean VAS score decreased to 1.12, the AOFAS score increased to 92.53, and the KAFS score increased to 93.36. All patients returned to normal gait, without mechanical instability or symptomatic instability of the ankle. There is no need for revision surgery.

### *Surgical Skills and Directions*

Arthroscopic anatomical repair of ATFL for CLAI is performed to achieve the expected therapeutic effect. It is crucial to note the following: (i) It is necessary to strictly grasp the inclusion and exclusion criteria, especially for patients with abnormal force line of the lower extremities. Simple repair of ATFL cannot achieve satisfactory results, and the postoperative recurrence rate is very high. (ii) The hyperplastic synovium tissue around the ankle and peroneal tip needs to be cleaned to reduce tissue swelling and pain. Before exploring ATFL, it is required that surgeons explore and deal with ankle lesions, especially a talus cartilage injury. (iii) The establishment of the anterior channel of the lateral malleolus under arthroscopy should be carried out in the safe area to avoid injury to the superficial peroneal nerve and sural nerve. (iv) The small bone mass of lateral malleolus avulsion can be removed to prevent the formation of corpus liberum and impact^29^. (v) During the operation, the position of the anchor should be located at the middle point of the footprint region of the distal fibula, and the bone freshening of the footprint region should be carried out before inserting the anchor to facilitate better healing of ATFL. The direction of the anchor should be at an angle of 30°–45° with the *y*‐axis of the fibula on the sagittal plane to prevent the distal end of the fibula from piercing and splitting^30^. In the horizontal plane, the anchor should be located at the midpoint of footprint to prevent the anchor from penetrating into the articular cavity or into the lateral cortex of the fibula. (vi) The length and quality of ATFL should be determined during the operation. If the repair conditions cannot be satisfied, the ligament should be reconstructed or the approach should be changed to that of the Broström‐Gould procedure^31^. (vii) Strength of ATFL fixation should be confirmed under the arthroscopy before ring suture and tight suture to prevent tear or fixation failure of ATFL during the suture process. (viii) When using the suture hook to suture the ring of ATFL, it is necessary to select a position of good quality to prevent the tear of ATFL in the process of stitching and knotting. (ix) Knotting should be carried out under the supervision of arthroscopy to ensure the intensity and effect of ligament suture. It is required to do standardized rehabilitation exercises to prevent joint stiffness and varus limitation.

### *Surgical Advantages*

Although the traditional open operation can accomplish direct ATFL repair, it cannot be used to deal with lesions in the joint, such as cartilage injury or the hyperplasia of synovium. Compared with the traditional open surgery, arthroscopic surgery has the following advantages: (i) There is a small incision, without wound complications, which can greatly evaluate the situation of the ankle and ATFL. Besides, accurate treatment can be performed. (ii) The lateral joint capsule of the ankle cannot be injured in the procedure, protecting the proprioceptor on the ankle capsule, which is beneficial to the recovery of proprioceptive function after the surgery. (iii) Nutritional vessels on ATFL are protected in arthroscopic surgery, which is beneficial for the recovery of the ligament and tendon–bone healing after surgery^32^. (iv) The anatomical repair of ATFL is realized without affecting the range of motion of the ankle and subtalar joint. (v) Operating in the safe zone is preferred as it does not affect the mobility of the ankle and the subtalar join^33^. (vi) Suture intensity and fixation effect of ATFL can be observed under the arthroscopy. (vii) Hospital stays are short and function recovers well. The average postoperative hospital stay of 37 patients was only 3.77 days, which was significantly shorter than that of traditional postoperative in‐hospital recovery^34^. Regarding the therapeutic effect on this group, the ankle pain disappeared completely, and the ankle‐inverted stress tests were negative. The anterior drawer tests of the ankle were negative. The range of motion of the patients was good. All patients returned to normal gait. There was no mechanical instability or symptomatic instability of the ankle.

### *Limitations*

There are, however, some limitations to this procedure: (i) It requires the operator to have a good arthroscopic technique and related anatomical knowledge; (ii) The criteria of anatomical repair or ligament reconstruction or superimposed suture of extensor retinaculum in ATFL were mainly based on varus stress test, drawer test, and operator experience, but there was a relative lack of objective indexes; (iii) This study is a retrospective study and thus lacked a control group. However, well‐designed prospective comparative studies are still needed to further confirm the long‐term functional outcomes of this procedure.

### *Conclusions*

Arthroscopic anatomical repair of ATFL for CLAI can achieve accurate ligament anatomical repair. The surgical trauma is small and the effect is reliable. In addition, the medium‐ and long‐term function is satisfactory, and therefore it can be applied extensively in the clinic.
